# Current Challenges and Opportunities for Improved Cannabidiol Solubility

**DOI:** 10.3390/ijms241914514

**Published:** 2023-09-25

**Authors:** Khondker Rufaka Hossain, Amani Alghalayini, Stella M. Valenzuela

**Affiliations:** 1 School of Life Sciences, University of Technology Sydney, Sydney, NSW 2007, Australia; khondker.hossain@uts.edu.au (K.R.H.); amani.alghalayini@uts.edu.au (A.A.); 2 ARC Research Hub for Integrated Device for End-User Analysis at Low-Levels (IDEAL), Faculty of Science, University of Technology Sydney, Sydney, NSW 2007, Australia

**Keywords:** bioavailability, cannabidiol, CBD, solubility, stability

## Abstract

Cannabidiol (CBD), derived from the cannabis plant, has gained significant attention due to its potential therapeutic benefits. However, one of the challenges associated with CBD administration is its low bioavailability, which refers to the fraction of an administered dose that reaches systemic circulation. This limitation necessitates the exploration of various approaches to enhance the bioavailability of CBD, thus helping to maximize its therapeutic potential. A variety of approaches are now emerging, including nanoemulsion-based systems, lipid-based formulations, prodrugs, nanocarriers, and alternative routes of administration, which hold promise for improving the bioavailability of CBD and pave the way for novel formulations that maximize the therapeutic potential of CBD in various medical conditions. This opinion piece presents the current understanding surrounding CBD bioavailability and considers strategies aimed at improving both its absorption and its bioavailability.

## 1. Introduction

Cannabidiol (CBD) is a phytocannabinoid extracted from cannabis plant species, including *Cannabis indica*, *Cannabis sativa*, or *Cannabis ruderalis* (also commonly known as hemp or marijuana) [1]. Although structurally similar to the psychoactive cannabinoid delta-9-tetrahydrocannabinol (THC), CBD does not cause intoxication or euphoric states and thus has low abuse potential while exhibiting a wide range of pharmacological effects [2]. A number of studies and clinical trials are now emerging that show CBD exhibits various therapeutic effects in conditions that range from epilepsy, psychotic disorders, anxiety, diabetes, sleep disorders, cardiovascular diseases, rheumatoid arthritis, pain, skin aging, antioxidant, and inflammation to cancer therapy [3,4,5]. As the therapeutic virtues of CBD are becoming better known and accepted, and given its favorable safety profile, a large number of countries globally have now legalized the use of CBD for medicinal purposes [6].

Studies suggest that CBD’s molecular activities are via the human endocannabinoid system (ECS), which includes two main cannabinoid receptors (CB1 and CB2) and endogenous ligands called endocannabinoids known to modulate CB receptor activities [7,8,9]. CB1 receptors are predominantly found in the central nervous system (CNS) and are highly expressed in regions such as the cerebral cortex, basal ganglia, hippocampus, and cerebellum. CB1 receptors are also present in peripheral tissues like the heart, liver, pancreas, muscles, adipose tissue, and the reproductive system. CB2 receptors are mainly expressed in cells related to the immune system, such as leukocytes, and are also found in the spleen, thymus, bone marrow, and other tissues related to immune functions. Although the therapeutic benefits of CBD are mainly generated from CBD’s role in the ECS, CBD does not directly activate the cannabinoid receptors instead, it has been shown to influence endocannabinoid balance [9]. There are two endocannabinoids, anandamide (AEA) and 2-arachidonoylglycerol (2-AG), that act as ligands for the cannabinoid receptors, and degradation of these endocannabinoids by fatty acid amide hydrolase (FAAH) and monoacylglycerol lipase (MAGL) enzymes has been shown to regulate CB receptor activities. CBD by inhibiting FAAH enzymatic activity has been shown to increase endogenous levels of AEA, which in turn has been shown to modulate the CB receptors, thereby indirectly exhibiting a wide range of pharmacological effects (for further details, see review [9]).

**Figure 1:**
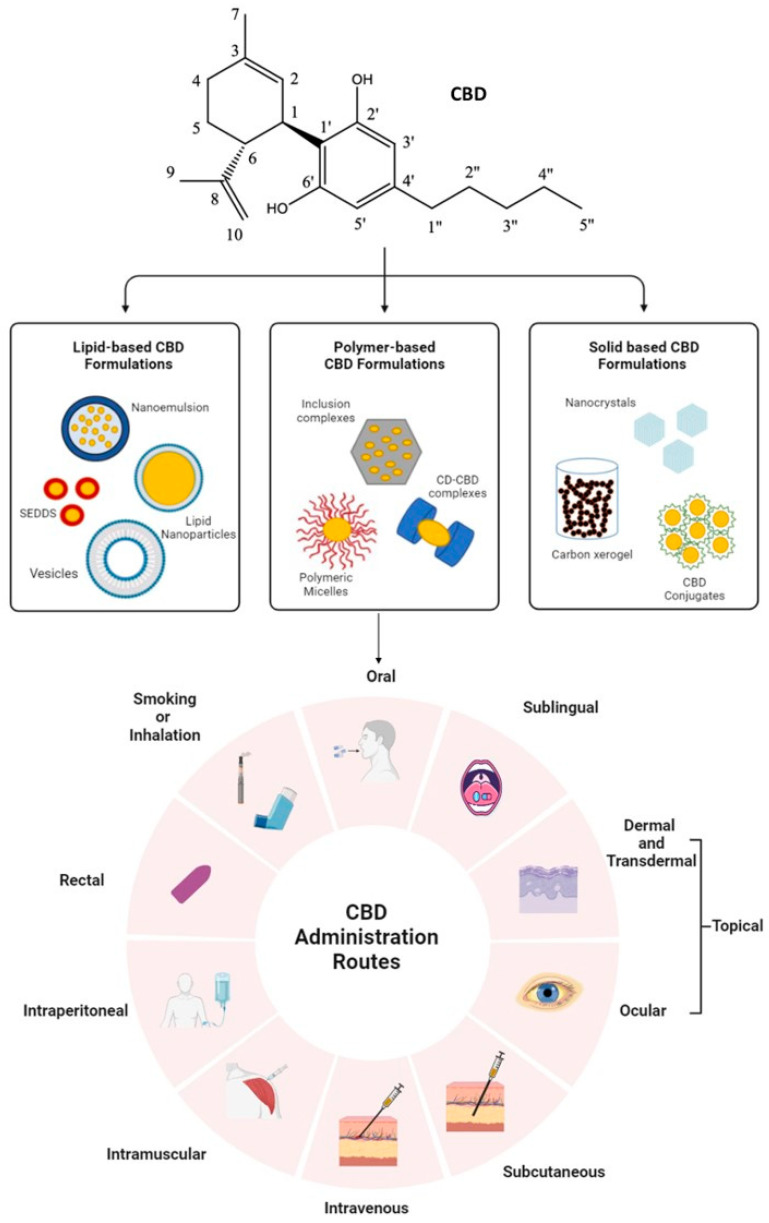
An illustration summarizing the range of different chemical modification or encapsulation approaches currently being examined in order to enhance CBD solubility, along with a variety of administration routes (Image created on Biorender.com).

In addition, CBD also binds with varying affinity to a series of other receptors, including but not limited to transient receptor potential vanilloid (TRPV), peroxisome proliferator-activated receptor gamma (PPARγ), as well as G protein-coupled receptors like GPR55 and serotonin 1A receptor (5-HT1A). CBD has allosteric binding activity with these receptors, where CBD binds to CB1 as an inverse agonist/antagonist; its binding to CB2 and GPR55 sees it acting as an antagonist; and on TRPV receptors and 5-HT1A, it acts as a partial agonist, respectively. It is also an inhibitor of the FAAH enzyme, and inhibition of FAAH and its interaction with 5-HT1A and TRPV1 receptors were found to play a role in CBD’s antipsychotic properties. The anti-depressive and anxiolytic activities of CBD were also attributed to its interaction with 5-HT1A. CBD has also shown high affinity for receptors and channels related to epilepsy, including TRPV receptors, T-type Ca^2+^ channels, serotine receptors, and GPR55. Studies have also shown that by inhibiting FAAH enzymes and GABA receptors, CBD is able to influence sleep. On the other hand, suppression of IFN-γ and TNF-α production and inhibition of T-cell proliferation by CBD were attributed to its role in diabetes. The affinity and action of the CBD-related receptors and the molecular mechanisms of action of the therapeutic effects of CBD within different disease contexts have been summarized in detail by Peng, J. et al., 2022 [9]. Furthermore, CBD has also been shown to exhibit antioxidant properties not by interacting with any receptors or enzymes but simply due to the presence of two hydroxyl groups in its chemical structure (Figure 1) that endow it with antioxidant activity [10].

To date, several CBD-based drugs have been approved by the United States Food and Drug Administration (FDA), the European Medicines Agency (EMA), the Australian Therapeutic Goods Administration (TGA), and other regulatory agencies worldwide [11]. Some examples of approved CBD-based drugs include Epidiolex, which is currently approved by the FDA as an oral solution and primarily prescribed for managing seizures associated with Lennox–Gastaut syndrome or Dravet syndrome in patients 2 years of age and older [12]. Arvisol is another patented oral tablet that contains pure, natural CBD and is recommended for the treatment of Rett syndrome, schizophrenia, and epilepsy. Several other CBD-based medications are also currently undergoing clinical or pre-clinical trials for their therapeutic application in conditions such as those mentioned above; for details, see the review by Stella, B. et al., 2021 [13]. In addition, due to its antioxidant capabilities, there are now more than a few CBD-based skincare products also marketed in the form of oils, gummies, capsules, and even creams [10].

Nevertheless, even though CBD is increasingly being successfully used as a therapeutic agent and for assisting in the management of several conditions, future CBD-based medications and clinical applications remain subject to a number of limitations and challenges. Low oral bioavailability of CBD remains one of, if not the most challenging, issues posing a hindrance to the further success of these compounds as therapeutic agents. This opinion piece therefore aims to highlight some of the challenges associated with the poor bioavailability of CBD molecules, with the objective of presenting some of the current strategies being explored to overcome these challenges. To compile a comprehensive opinion piece, an extensive search of the literature was conducted on relevant databases like PubMed, Scopus, Science Direct, and Web of Science. Only peer-reviewed journal articles and already patented formulations/methods claiming to improve the solubility and/or bioavailability of CBD were included. Also, articles not dating back to more than 5–10 years were selected in order to highlight recent and relevant research. Older articles were only included if they were seminal or had biological significance.

## 2. CBD Chemical Structure, Absorption, and Bioavailability

CBD is a 21-carbon terpene phenolic compound, thus making it a relatively large molecule. Its chemical structure is composed of a long linear hydrocarbon chain with a benzene ring at one end, as shown in Figure 1. This structure gives it a large hydrophobic area, which contributes to its lipophilicity and hydrophobicity [14]. Thus, CBD’s poor water solubility and high lipophilicity result in its relatively low and inconsistent bioavailability [15].

As a hydrophobic molecule, CBD is primarily absorbed through passive diffusion in the gastrointestinal tract. After oral administration, CBD interacts with bile salts and forms micelles, which facilitate its absorption by the small intestine [16]. The absorption of CBD can be influenced by factors such as formulation, food intake, and individual variations. In addition, evidence of its interaction and effects on the gastrointestinal microbiota is now emerging, which may also influence its absorption and activity (for further details, see the following review papers [17,18,19]). Once absorbed, CBD, being hydrophobic, binds to plasma carrier proteins, particularly lipoproteins and albumin, for transportation throughout the body. Although the search for intracellular transporters for phytocannabinoids is still being extensively researched, studies have shown fatty acid-binding proteins (FABPs) act as soluble intracellular carriers for the transportation of these hydrophobic compounds from the plasma membrane to the site of action [20]. FABPs are intracellular proteins that mediate AEA transport to its catabolic enzyme FAAH, primarily localized in the endoplasmic reticulum [21]. CBD has been shown to bind soluble FABPs for transportation to FAAH enzymes, and binding of CBD to FABPs has also been shown to inhibit transportation of AEA, resulting in reduced catabolism of AEA by FAAH [20,21]. Being lipophilic, CBD also tends to accumulate in lipophilic tissues such as adipose tissue, further contributing to its low bioavailability [22]. Despite these absorption obstacles and poor bioavailability, one of the advantages of CBD is that it can cross the blood–brain barrier and appears not to be a substrate for P-glycoprotein, thus making it an interesting drug for central nervous system applications [23]. Another advantage of CBD is that not only can it be structurally modified but also successfully formulated into various different dosage forms for administration via different routes, which presents a plethora of possibilities to overcome these obstacles. The following section highlights some of the factors affecting the bioavailability of these compounds and the delivery approaches being trialed in order to improve the bioavailability and solubility of CBD for enhanced therapeutic benefits.

## 3. Factors Affecting the Bioavailability of CBD

Physiochemical properties of CBD, like solubility and stability, along with its bioactivity, permeability, and metabolism, are some of the main elements that affect the bioavailability and absorption rates of CBD compounds, as well as variable pharmacokinetic profiles and possible polymorphisms. Studies have shown polymorphisms to significantly impact the behavior of the CBD molecule and, in turn, its potential therapeutic activity. CBD presents in two or more inherent crystalline forms that can affect its stability, which is a concern as this, in turn, influences CBD’s absorption rate and thus its bioavailability [24]. CBD is also known to be sensitive to light [25], temperature [26] and auto-oxidation [10]. Mazzetti, et al. (2020) demonstrated that CBD samples stored in the dark showed less degradation compared to CBD samples exposed to light [25]. CBD is also sensitive to heat, where incubation of CBD compounds under high-temperature conditions for long periods of time can reduce CBD stability [26]. Furthermore, oxidation of CBD has been shown to contribute to its degradation, thus altering the pharmacological properties of CBD and reducing its potential therapeutic benefits, as most of it would be broken down before it reaches the bloodstream [10]. Although the greatest limitation in CBD drug development comes from the poor solubility of these compounds, another major factor that affects CBD’s bioavailability is that it undergoes extensive first-pass metabolism when taken orally [27,28].

After oral administration, the portion of the CBD that is absorbed first travels to the liver via the hepatic portal system, where it undergoes extensive first-pass metabolism [27,28] primarily by cytochrome P450 (CYP) enzymes and specifically the CYP3A subfamily [29]. These enzymes convert CBD into various metabolites, the main ones being 7-carboxy-cannabidiol (7-COOH-CBD), 7-hydroxy-cannabidiol (7-OH-CBD), and a minor metabolite called 6-hydroxy-cannabidiol (6-OH-CBD). Among the identified major metabolites of CBD in humans, the most abundant metabolite, 7-COOH-CBD, was found to be inactive, which further impacts the bioavailability of these compounds [15]. The remainder is then transported in the blood to the site of action. Since the effectiveness of any CBD medication depends primarily on its successful delivery and uptake at the intended site of action, increasing efforts are being made to improve the bioavailability of cannabinoids. Hence, different routes of administration that can help bypass the first-pass hepatic metabolism are constantly being investigated in order to improve bioavailability [27,28].

## 4. Administration Routes of CBD

The choice of administration route can significantly impact the bioavailability, onset of action, and overall effectiveness of CBD. There are a variety of different administration routes, for example, oral, sublingual, dermal and transdermal, topical, subcutaneous, rectal, intramuscular, intraperitoneal, and smoking or inhalation, that have been implicated in improving CBD bioavailability.

Administration of CBD via the oral route is the most common and convenient for patients and involves the ingestion of CBD products such as capsules, edibles, or tinctures [30]. However, it is associated with several drawbacks. Firstly, CBD being hydrophobic results in low absorption of the drug, and what is absorbed is subjected to extensive first-pass metabolism, resulting in a low and variable reported oral bioavailability of approximately 9–13% [4,14]. Secondly, the oral route’s onset of action is delayed compared to other routes, typically ranging from 30 min to 2 h [15]. To overcome this problem, studies have suggested oral consumption of CBD with food with high lipid content or in a lipid solution, which has been shown to enhance CBD bioavailability [3,4,31].

In addition, other routes of administration have also been investigated that have been shown to improve CBD bioavailability. One such route is the sublingual administration of CBD. This route allows CBD oil or extract to be absorbed directly into the bloodstream through the sublingual mucosa, which helps to bypass first-pass metabolism. Studies have shown CBD administered through the sublingual route results in a higher bioavailability of approximately 12% to 35% compared to the 9–13% observed for oral administration [32,33]. Currently, orally disintegrating tablets, or orodispersible (ODTs) CBD tablets, have also been developed by several different methods with varying formulations for sublingual administration, as these tablets are designed and formulated to disintegrate directly in the mouth for absorption [34,35,36]. ODTs also have the advantage of delivering large amounts of CBD that increase bioavailability, mainly due to the ease of manufacturing, where CBD can be compressed directly as solid or liquisolid powdered tablets [34,35,36]. In a study aimed at developing ODTs with CBD using varying formulations, an optimal formulation had a disintegration time of 27 s and 99.3 ± 6% of CBD released within 30 min [36]. Another study showed CBD–Ethanol liquisolid ODTs to exhibit a similarly high dissolution efficiency of 93.5 ± 2.6% [34].

However, the highest concentration of CBD in the blood was found using an intravenous injection method, followed closely by inhalation via smoking, which facilitated rapid delivery of CBD [3,4,37]. Inhalation of CBD involves vaporizing CBD oil or smoking CBD by using a vaporizer or traditional smoking methods, and these appear to offer some of the highest bioavailability among all administration routes, approximately 31–45%. However, studies have shown that vaporizing or smoking CBD has the effect of shortening the half-life of CBD in the blood [4,33]. In addition, a recent study showed that vaping CBD induces a potent inflammatory response with higher oxidative damage, leading to increased pathological changes associated with lung injury compared to vaping nicotine [38]. Hence, there is a pressing need for further investigation and for alternative routes or strategies to improve CBD solubility and bioavailability.

Other routes that have also been investigated include dermal and transdermal administration. This route involves applying CBD-infused products, such as creams or lotions, directly to the skin, enabling CBD to penetrate through the skin layers and reach the systemic circulation. However, both dermal and transdermal routes offer limited bioavailability (1% to 10%) [33,39], and several studies reported the accumulation of CBD in the outermost layers of the epidermis without penetration to the deeper tissue layers (see review articles [4,11,14,40]). Another topical application involving the ocular administration of CBD, primarily for conditions such as glaucoma or inflammation, has also shown limited systemic absorption, with CBD predominantly acting locally within the eye [41]. Although topical administration routes target specific areas of the body, providing limited systemic absorption and localized relief, particularly for conditions such as inflammation, arthritis, and muscle pain, these routes are still associated with low bioavailability [37].

Evaluation of additional routes, such as the subcutaneous administration route where CBD was injected into the fatty tissue layer beneath the skin, has been shown to provide a slow and sustained release of CBD into the systemic circulation and bypass the liver first-pass metabolism [42,43]. Similar advantages were also provided by the intramuscular administration route, which involves injecting CBD directly into the muscle tissue [44]. However, these routes are associated with a delayed onset of action due to the slow release of the compound into the circulation. A current study aimed at investigating the intraperitoneal administration of CBD using nanoparticle delivery systems for ovarian cancer treatment showed rapid absorption of CBD into systemic circulation and provided high bioavailability [45], but this has the disadvantage of being an invasive procedure. Recently, rectal administration of CBD, in the form of suppositories or enemas, has been shown to be a very promising route, as it not only bypasses first-pass metabolism but also offers higher bioavailability in the range of 13% to 50% in comparison to oral administration [4,38]. Yet, there remains a lack of detailed studies, clinical trials, or preclinical trials examining the pharmacokinetics of CBD when administered rectally, and one cannot ignore the discomfort associated with rectal administration.

Despite several drawbacks, pharmaceutical-grade, regulated cannabinoid-based medicinal products are increasingly becoming readily available for prescription worldwide, either as an oily or alcoholic formulation, in soft-gel capsules, liquid solutions, sublingual drops or tablets, or as oromucosal sprays [11]. However, they still only offer limited bioavailability and are associated with poor solubility and stability. These present major difficulties in designing and systemically delivering cannabinoid-based formulations for most therapeutic applications. As such, a variety of studies aimed at developing new CBD formulations to overcome the physicochemical limitations of the molecule by delivering it through different routes remain ongoing [13,46]. In the meantime, alternate approaches are emerging, which are now aiming to improve and modulate drug solubility by using advanced carriers. Some of these strategies and approaches are highlighted and discussed below.

## 5. Approaches to Improve CBD Solubility and Bioavailability

Several techniques have been utilized to improve the drug’s dissolution profile, reduce its degradation, and promote and control its site-specific release, all in order to improve CBD bioavailability. One such approach involves the incorporation or complexing of CBD with advanced carriers in order to formulate amorphous solid dispersions, polymer-based CBD inclusion complexes, lipid-based formulations, and/or nanoformulations administered via different routes [13]. These CBD formulations have been shown to exhibit enhanced solubility profiles, which in turn facilitate CBD absorption, resulting in improved bioavailability. Figure 1 illustrates the different approaches currently being tried to enhance CBD solubility, including via a variety of administration routes.

## 6. Lipid-Based CBD Formulations

Increasingly, investigations are turning to the use of lipid-based formulations, which tend to enhance the solubility and delivery of various lipophilic drug molecules. There are now numerous studies of CBD lipid-based formulations where CBD has been encapsulated in macro- or nano-structured lipid carriers like lipid nanocapsules, liposomes, vesicles, or loaded into self-emulsifying drug delivery systems (SEDDS) as nanoemulsions and/or as microemulsions to increase water solubility [11,47]. Distinct lipid nanocapsules with CBD located either in the oily core or at the surface have shown a 3.4-fold increase in solubility and permeability across the blood–brain barrier in comparison to CBD alone, due to their small particle size (20–55 nm), and have been implicated in treating gliomas in the brain [47,48,49]. Studies using vesicular CBD delivery systems also showed enhanced solubility and bioavailability. A vesicular lipid–CBD system termed ethosome, composed mainly of phospholipids, ethanol, and water, showed enhanced permeability through the skin, resulting in a 40% CBD plasma concentration after transdermal application in comparison to the 1–10% general seen from transdermal or dermal application of CBD [50]. Additionally, cardanol (a compound that acts as a co-surfactant when combined with cholesterol), when used as a solvent for CBD, has been shown to produce self-assembled nano-vesicular systems with enhanced aqueous solubility [51].

Other lipid-based methods to increase oral CBD bioavailability include self-emulsifying drug delivery systems (SEDDS). These are isotropic mixtures of oils, surfactants, and co-solvents that tend to emulsify into macro- or nanodroplets in an aqueous medium, such as in the gut, thereby providing in situ drug solubilization. The small nature of the droplets increases the surface area available for drugs to be dissolved and absorbed, thereby increasing both the solubility and bioavailability of cannabinoids [14,52,53,54]. For example, a patented oral CBD-based formulation named PTL101, manufactured using proprietary gelatin matrix pellets, demonstrated greater bioavailability of about 31–34% compared to a reference oromucosal spray in humans [55,56,57]. VESIsorb^®^ (Baar, Switzerland) is another novel lipid-based delivery system that self-assembles into a colloidal delivery system on contact with water, resulting in a 4.4-fold increase in CBD plasma concentration [58]. Echo Pharmaceuticals and Ananda Scientific are also investigating formulations using their patented lipophilic compound delivery technology to increase CBD’s water solubility. Currently, both compounds are in preclinical or clinical development phases [14]. Several other types of CBD nano- and microemulsions have also been developed using different oily mediums like soybean oil, sesame oil, and triglycerides with/without stabilizers, resulting in a high CBD load observed for all the emulsions in comparison to CBD alone [40,47,59].

## 7. Polymer-Based CBD Inclusion Complexes

Polymer-based CBD inclusion complexes, especially those produced using biodegradable polymers, have attracted attention as promising advanced drug carriers to enhance CBD solubility, bioavailability, stability, and different drug release profiles.

CBD inclusion complexes formed using two different biodegradable polymers, (poly-lactic-co-glycolic acid) (PLGA) and poly-ε-caprolactone (PCL) showed significantly different particle sizes and release profiles. Each polymer interacted differently with the release medium, leading to different degradation routes and rates, which further resulted in different drug diffusion times and profiles [45,47,60,61,62]. Hernán Pérez de la Ossa et al. (2012) developed spherical CBD-loaded poly-ε-caprolactone microparticles with a size range of 20–50 nm and high CBD entrapment efficiency to improve bioavailability by allowing slow release of the drug over a period of 10 days [62]. PLGA–CBD inclusion nanocomplexes coated with chitosan were also developed that showed significantly higher absorption after 2 and 6 h of incubation, resulting in higher bioavailability [40,63]. Rao et al. (2022) showed encapsulation of CBD in Poloxamer 407 (P407), which is a triblock copolymer composed of (poly)ethylene oxide and (poly)propylene oxide sections, results in the formation of self-assembled nanomicelles with improved bioavailability [64]. In a further study, zein/whey protein nanoparticles loaded with CBD showed a significantly increased solubility of ca. 196 μg/mL in comparison to 12.6 μg/mL observed for CBD. The zein/whey protein–CBD nanoparticles also showed protection from CBD degradation by heat and UV light, thereby providing excellent storage stability [65].

Another approach to increasing solubility includes the use of cyclodextrins (CD) to carry lipophilic molecules by means of complexation methods [47,66,67]. Cyclodextrins can accommodate small organic molecules like CBD within their lipophilic central cavity, resulting in water-soluble CBD inclusion complexes with an apparent solubility of up to 5000 μg/mL. Several studies have also investigated the efficacy of different isoforms of cyclodextrin, such as α-CDs, β-CDs, and γ-CDs [47,66,67,68,69,70,71,72]. The water solubility of CD–cannabinoid complexes can be further improved by chemically derivatizing β-CD. AOP Orphan Pharmaceuticals significantly increased the aqueous solubility and bioavailability of the drug by using randomly methylated β-CD and hydroxypropyl-β-CD in a patented technology [11,73]. Medexus pharmaceuticals and Vireo Health LLC have developed two proprietary formulations of CBD and cyclodextrins with improved CBD solubility and bioavailability and are currently undertaking clinical trials [14]. Another patent application has been filed for the complexation of cannabinoids with sulfo-alkyl-β-CD in the presence of Cremophor EL (polyoxyl-35 castor oil) to promote cannabinoid solubility [74]. Furthermore, CBD–cyclodextrin complexes have the advantages of being cost-effective, easy to produce, and can be delivered in a solid-state dosage form with improved stability and shelf-life [47].

## 8. Solid-Based CBD Formulations

CBD nanocrystals, CBD on carbon xerogel microspheres, and CBD conjugates are examples of other approaches currently being explored to improve CBD solubility. CBD nanocrystals were developed to improve the stability, bioavailability, and therapeutic effects of CBD. A patent by Dickman D and Levin D (2017) describes a novel CBD crystalline form with increased aqueous solubility due to its lower melting point temperature [75]. Furthermore, solid CBD nanocrystals can help improve the bioavailability of the compounds as they can allow 100% of the drug to reach the gastrointestinal tract. BeneCeed™, a 200 mg CBD tablet by Columbia Care, is now being assessed under a UK clinical trial [11]. A randomized, open-label crossover trial is underway in Australia for two patent applications, filed by AusCann Group Holdings Ltd., Perth, Australia, for solid compositions containing either a single or a blend of CBD, medium-chain triglycerides, surfactants, and colloidal anhydrous silica [76,77]. Axim Biotech Inc., New York, NY, USA, has developed a controlled-release chewing gum containing a 1:1 combination of CBD and THC to treat patients with multiple sclerosis-related pain and spasticity, Parkinson’s disease, dementia, restless leg syndrome, and post-herpetic neuralgia [78,79]. A patent by GW pharmaceuticals lists solid-state CBD as a potential clinical consideration in the treatment of inflammatory bowel disease [14,80]. Echo Pharmaceuticals has also developed a CBD pill formulation (Arvisol^®^, Leiden, The Netherlands) with improved bioavailability to treat neurological conditions, including Rett syndrome, schizophrenia, and epilepsy [81,82]. Furthermore, a dry, compressed cannabis inhalable formulation developed by Tetra Bio-Pharma, Orleans, ON, USA, is currently being tested in clinical trials in Canada and the USA [11].

Carbon xerogel microspheres are also claimed to provide an excellent carrier system for CBD due to their high purity, multiple controlled forms with well-developed and fitted porosity, and excellent surface chemistry [83,84]. A number of CBD gel formulations by Botanix pharmaceuticals are in early clinical development for transdermal applications to treat acne, psoriasis, and dermatitis [85,86,87]. Formulations based on CBD–carbon xerogel microspheres showed enhanced bioavailability when implemented for both oral and intranasal applications [14]. Zynerba Pharmaceuticals has also developed a permeation-enhanced CBD gel, “Zygel”, for transdermal applications that is now in phase II clinical trials [88].

CBD conjugation is another useful method for overcoming problematic properties of the drug by increasing the bioavailability, solubility, dissolution rate, physical form, melting point, tableting, stability, or permeability of CBD complexes [11,14,47]. For example, TurboCBD^TM^ capsules developed by Lexaria Bioscience Corp. (Columbia, CA, USA) showed that association of CBD with long-chain fatty acids allowed higher concentrations of CBD to enter the circulatory system, resulting in a plasma concentration of 80–85% by 90 min [89]. Harris et al. (2019) showed conjugation of CBD with poly(2-oxazoline) connected via releasable linkages, resulting in a significantly slower hydrolytic release of the cannabidiol than the corresponding PEG–CBD and dextran–CBD conjugates [90]. Artelo Biosciences has developed a CBD–tetramethylpyrazine conjugate, ART12.11, that is currently in the nonclinical phase of pharmaceutical development targeted towards post-traumatic stress disorder (PTSD), inflammatory bowel disease (IBD), stroke, and rare diseases. This CBD–tetramethylpyrazine conjugate offered increased efficacy and bioavailability by acting synergistically and changing the physiochemical properties that are associated with ineffective absorption [14]. Claritas Pharmaceuticals, Seattle, WA, USA, is developing an oral CBD/naproxen combination drug that targets the spinal cord to treat acute and chronic pain. They are also working on an intravenous (IV) formulation of the CBD/naproxen drug [11]. Preclinical trials using CBD conjugates pioneered by Diverse Biotech Inc. (Orlando, FL, USA) are also currently underway for the treatment of cancer [91]. In addition to these approaches, water-soluble CDB derivatives like L-valine-ester or bi-sulphate CBD derivatives have also been developed by Kalytera for different routes of administration [92]. It is important to note, however, that many of the approaches to enhancing CBD bioavailability highlighted above still require consideration of the mode of administration, CBD extraction process, manufacturing techniques, product formulations, pharmacokinetic profiles, and ultimately the targeted therapeutic application.

## 9. General Discussion

Over the past several decades, numerous strategies and CBD formulations have been explored, such as route of administration, medium of administration, conjugation, and structural modifications of CBD itself, in order to overcome the challenges of low bioavailability. Several of these strategies and formulations have been highlighted in this opinion piece. We have seen how different encapsulation approaches, such as micro/nanoemulsions, dendrimers, liposomes, micelles, biodegradable polymer particles, and nano-structured lipid carriers, both at the nano- and micro-scale platforms, have been formulated using various strategies to improve the solubility and bioavailability of CBD to varying degrees. The use of long-chain triglycerides such as sesame oil, cocoa butter, tricaprin, or lipids from food has also been shown to improve CBD bioavailability, particularly by contributing to the lymphatic absorption of these cannabidiols [11]. Previously, the use of lipids has proven valuable for increasing the bioavailability of lipophilic drugs, such as cannabinoids. This, however, is greatly dependent on the type and length of the lipid chain, degree of saturation, and digestibility of the oily excipients, which may all influence the oral absorption and bioavailability of CBD, resulting in variable outcomes [93]. The use of several different adjuvants, like surfactants, solubilizers, cosolvency, hydrotrophy, and novel excipients, alone or in combination, has also been shown to improve solubility [11]. The use of nonionic surfactants like macrogolglycerol hydroxystearate to form CBD micelles demonstrated a two-fold increase in CBD bioavailability. But potential drawbacks of using adjuvants include the need to use significant amounts of these compounds, which might lead to irritation within the gastrointestinal tract. There is also the possibility of drug leakage from capsules and stability concerns, including the potential migration of co-solvents and drug precipitation during storage [93]. Polymeric encapsulation of CBD in PLGA and surface modification using coating agents like vitamin E, lecithin, chitosan, and PEG–chitosan increased encapsulation efficiency [45,60]. Despite their potential, there are certain drawbacks associated with the utilization of polymeric nanoparticles. These drawbacks pertain to their constrained shapes, chemical composition, broad size distribution, tendency to aggregate, and electromagnetic characteristics, all of which can result in issues such as limited absorption when taken orally, instability within the bloodstream, and insufficient dispersion in tissues. While most polymeric nanoparticles tend to be spherical in shape, their synthesis can yield a diverse array of sizes, resulting in batch-by-batch inconsistency at the industrial scale [94].

Apart from different formulations, the use of diverse strategies for CBD delivery has also been implemented. For example, several nanoparticle-based CBD delivery systems consisting of nanoconjugated cannabidoils in a multifunctional metallic nanocarrier, either classified as inorganic, organic, or hybrid nanosystems, depending upon the specific requirements and administration route, are currently in preclinical or clinical development [11,13,74]. Furthermore, lipid-based CBD nanoformulations are also a promising strategy; however, self-emulsifying CBD delivery systems (SEDDS) have become a more lucrative technique to improve CBD bioavailability. A study showed that the addition of natural absorption enhancers like curcumin, resveratrol, and piperine to SEDDS can further increase CBD oral bioavailability in vivo, with piperine having the maximum effect [76]. Despite the implementation of several new and novel approaches, its lipophilic nature, low bioavailability, and excessive first-pass metabolism still remain as challenges that largely impact access to CBD-based therapeutics.

In recent years, structural modification or complexation of CBD with cyclodextrins or carbohydrates has also shown promising results, where further structural and chemical modification of the cyclodextrins can greatly increase CBD solubility and hence bioavailability. Structural modification of several other lipophilic drugs by complexation with cyclodextrins or modified carbohydrates to enhance solubility has been successfully utilized and is now marketed worldwide for their therapeutic effects (for details, see reviews [95,96,97,98]). These complexes also have the ability to be formulated for delivery through various routes of administration, thus giving them an added advantage over other techniques [95,96,97,98]. Complexation does have definite advantages; however, proof that complexation does not alter the physiochemical and therapeutic characteristics of the drug remains to be confirmed and warrants further investigation.

## 10. Conclusions and Future Perspectives

CBD has gained significant attention for its potential therapeutic and medical benefits. Poor bioavailability, however, remains one of the significant obstacles to CBD’s development and application for these purposes. Increasing bioavailability by improving CBD solubilization in the aqueous phase appears to be the main current focus. Utilizing technologies like nanoemulsion and liposomal delivery, co-administering with healthy fats, exploring sublingual administration, and considering inhalation or solubility enhancement methods, along with structural modification and/or complexation of the compounds, have all been shown to significantly improve their bioavailability. Future research will no doubt focus on further optimizing CBD formulations in order to overcome their physiochemical limitations. These include exploring new carriers, understanding the impact of different administration routes on bioavailability, and conducting thorough pharmacokinetics studies. In addition, long-term safety studies and clinical trials are needed to evaluate the efficacy and safety of CBD formulations with improved bioavailability across a variety of medical conditions. Although the field of CBD research is moving rapidly, it remains to be seen which of these various approaches will provide the best outcomes for the delivery of CBD and its positive short- and long-term effects and efficacy.

## Data Availability

Not applicable.

## List of Abbreviations

CBD: Cannabidiol; THC, delta-9-tetrahydrocannabinol; ECS, endocannabinoid system; CB, cannabinoid receptors; CNS, central nervous system; AEA, anandamide; 2-AG, 2-arachidonoylglycerol; FAAH, fatty acid amide hydrolase; MAGL, monoacylglycerol lipase; TRPV, transient receptor potential vanilloid; PPARγ, peroxisome proliferator-activated receptor gamma; 5-HT1A, serotonin 1A receptor; FDA, United States Food and Drug Administration; EMA, European Medicines Agency; TGA, Australian Therapeutic Goods Administration; FABPs, fatty acid-binding proteins; ODTs, orally disintegrating or orodispersible tablets; SEDDS, self-emulsifying drug delivery systems; PGLA, poly-(lactic-co-glycolic acid); PCL, poly-ε-caprolactone; P407, Poloxamer 407; CD, Cyclodextrins.
